# Recruitment for Voluntary Video and Mobile HIV Testing on Social Media Platforms During the COVID-19 Pandemic: Cross-Sectional Study

**DOI:** 10.2196/54420

**Published:** 2024-11-28

**Authors:** Piao-Yi Chiou, Wei-Wen Tsao, Chia-Lin Li, Jheng-Min Yu, Wen-Han Su, Zhi-Hua Liu, Cheng-Ru He, Yu-Chun Chang, Yi-Hsuan Tsai

**Affiliations:** 1 School of Nursing, National Taiwan University College of Medicine Taipei Taiwan; 2 Department of Nursing, National Taiwan University Hospital Taipei Taiwan; 3 Taiwan AIDS Nurse Association Taipei Taiwan; 4 Taiwan Lourdes Association Taipei Taiwan; 5 Center for Neuropsychiatric Research, National Health Research Institutes Taipei Taiwan; 6 Institute of Health Policy and Management, College of Public Health, National Taiwan University Taipei Taiwan; 7 Department of Cardiovascular surgery, National Cheng Kung University Hospital Tainan Taiwan; 8 Department of traditional Chinese medicine, Kaohsiung Medical University Chung-Ho Memorial Hospital Kaohsiung Taiwan

**Keywords:** COVID-19, HIV testing, mobile health, risk-taking behavior, social media, video, mobile phone

## Abstract

**Background:**

The COVID-19 pandemic prompted social distancing policies and caused misinformation that hindered in-person HIV screening for high-risk groups. Social media platforms provide additional options for voluntary counseling and testing (VCT) for HIV, overcoming these limitations. However, there is a lack of data on HIV testing recruitment through social media platforms and its outcomes during the pandemic.

**Objective:**

This study aimed to measure the rate of face-to-face mobile and video VCT conducted after recruitment through social media platforms and friend referrals during the pandemic and compare the geographic distribution, risk feature targeting, testing outcome, and cost between the 2 models.

**Methods:**

Data were collected from March 3 to December 31, 2021, during the COVID-19 outbreak in Taiwan. Participants engaging in unprotected sex were recruited. After one-on-one message discussions through the platforms, the well-trained research assistants provided mobile or video VCT based on the participants’ availability. Primary outcomes were completion rate, testing results, and CD4 count. Secondary outcomes included demographic and HIV risk-taking and protective features from a questionnaire. Selection bias was controlled by adjusting for the testing site (Taipei vs non-Taipei) using univariable multinomial logistic regression.

**Results:**

This study gathered 5142 responses on the social media platforms, recruiting 1187 participants. Video VCT had a completion rate of 31.8% (207/651), higher than mobile VCT’s 21.8% (980/4491). Both rates were higher than those before the COVID-19 pandemic. Recruitment through friend referrals, instant messaging apps (eg, Line [LY Corporation]), and geosocial dating apps (eg, Hornet [Queer Networks Inc], Grindr [Grindr LLC], and Gsland [Tien-Hao Tsai]) resulted in higher acceptance and completion rates than social networks (eg, Facebook [Meta], X [formerly Twitter], and Instagram [Meta]). Mobile VCT had higher recruitment among urban residents and screening density, while video VCT reached a broader geographic area. The mobile group was more likely to have had more than 10 sexual partners (odds ratio [OR] 1.92, 95% CI 1.05-3.50; *P*=.03), history of sex work (OR 4.19, 95% CI 1.68-10.43; *P*=.002), and sexually transmitted diseases (OR 2.23, 95% CI 1.18-4.23; *P*=.01) within the past 3 months. The video group was more likely to meet sexual partners through social media. The HIV-positive rate in the mobile group was 0.7% (7/973) with an average CD4 count of 460/μL, while in the video group, it was 1% (2/205) with an average CD4 count of 347/μL, indicating a later diagnosis. Both positivity rates were higher than those before the COVID-19 pandemic, with no significant difference between the groups. The video group cost US $54.68 per participant, slightly higher than the US $50.36 for the mobile group.

**Conclusions:**

Recruiting through social media platforms that facilitate one-on-one message discussions can effectively target high-risk groups for mobile and video VCT. This approach should be integrated into the current screening model to enhance HIV case finding.

## Introduction

Although the number of confirmed cases and deaths associated with COVID-19 has gradually decreased, emerging infectious diseases continue to be a global public health concern [1-3]. In response to the COVID-19 outbreak, countries worldwide adopted prevention and control measures recommended by the World Health Organization to reduce virus transmission [4]. These measures included social and physical distancing mandates and implementing visitor control at hospitals and health care facilities [5], which directly or indirectly hindered the accessibility of face-to-face voluntary counseling and testing (VCT) services for HIV [6].

Studies have found that high-risk sexual behaviors and recreational drug use persisted or increased during the COVID-19 pandemic [7,8]. Despite lockdown policies, approximately 76% of the HIV target population, particularly men who have sex with men (MSM), continued sexual activities and 19% used recreational drugs owing to stress and sexual desires [9-12]. However, capacity for HIV testing was estimated to have dropped from 26% to 85% in Africa, America, Asia, and Europe; this reflected the situation pertaining to key populations who lacked access to VCT services during the pandemic [8,13-16]. Face-to-face mobile VCT distributes recruitment messages through social media platforms and offers flexibility in terms of testing locations and schedules for specific groups [17]. However, despite its high accessibility and convenience, service volume drastically decreased during the COVID-19 pandemic in Taiwan [18]. In contrast, home-based screening allowed participants to request a self-testing kit through postal services, with additional real-time counseling accessible internet, eliminating the need for face-to-face contact [19-23]. Therefore, implementing VCT for HIV through the video model provides an opportunity to adhere to social distancing guidelines and prioritize the safety of frontline screeners [14,24-28].

Social media platforms, including mobile instant messaging apps, geosocial network (GSN) apps using GPS, and social networks, serve as interactive digital channels. Considering the high ownership rate of smartphones in Taiwan [29], social media platforms, which have an expanding user base and lack face-to-face limitations, have emerged as effective channels for recruiting high-risk and hard-to-reach groups and maintaining uninterrupted VCT services [30-32]. The statistics on adult social media use in Taiwan indicate that the Line (LY Corporation) messaging app has the highest market share among instant messaging apps, accounting for a weighted value of 77.6% (2153/1670) [33]. The most popular social networks are Facebook (Meta), Instagram (Meta), and X (formerly Twitter), which cover 78.1% (16.95 million/21.71 million), 52.3% (11.35 million/21.71 million), and 21.4% (4.64 million/21.71 million) of the local internet user base, respectively [34,35]. Hornet (Queer Networks Inc), Grindr (Grindr LLC), and Gsland (Tien-Hao Tsai) are the most widely used GSN apps among lesbian, gay, bisexual, transgender, and queer (LGBTQ) users in Taiwan [36,37]. These platforms are popular for connecting with potential sexual partners among high-risk groups for HIV [38-40]. However, the use of social media platforms can vary due to discrepancies in internet infrastructure across different geographic areas. In rural areas of Taiwan, the personal internet access rate for individuals aged 12 years and older stands at 75.8%, which is 10.4% lower than the national average of 86.2% [41]. In addition, the COVID-19 infodemic, which refers to the spread of false and misleading information through social media platforms that causes confusion, risky behavior, and distrust toward health officials, might interfere with the recruitment of high-risk groups for HIV testing [42,43]. Existing data on participant recruitment and the outcomes of recruiting for HIV testing through social media platforms during the COVID-19 pandemic are scarce.

Therefore, this study aimed to evaluate the rate of face-to-face mobile and video VCT for HIV conducted after recruitment through social media platforms during the COVID-19 pandemic. It also examined the geographic distribution, targeted risk behavior features, testing outcomes, and CD4 counts to compare the health status of HIV-positive participants, as well as the cost between the groups tested using these 2 models.

## Methods

### Study Design

Data were collected from March 3 to December 31, 2021, during the COVID-19 outbreak in Taiwan. This study used a cross-sectional research design. Participants were selected using convenience and snowball sampling and recruited through popular social media platforms frequented by high-risk groups for HIV, based on data from the previous year (2020), as indicated by program CW108056. Participants were recruited through the Line messaging app, GSN dating apps, a fan page on Facebook, Instagram, and X. In total, 2 testing models, mobile and video VCT, were conducted by proficient research assistants according to the participants’ availability.

### Participants

The inclusion criteria were being aged 20 years or older, having the literacy skills to understand the research consent form and related questionnaires, and self-reporting engagement in unprotected sexual intercourse before a 3-month window period. The exclusion criteria were reporting no involvement in any unprotected sexual intercourse and having a previous HIV-positive identification.

### Procedures and Data Collection

#### Recruitment

For recruitment through Line, the QR code of the official account was disseminated across various platforms to encourage web-based users to engage in one-on-one discussions with our research assistants.

Recruitment through GSN dating apps was conducted through Hornet, Grindr, and Gsland. User profiles were refreshed every 2 hours during the daytime on weekdays to display our official account with the heading and profile describing HIV testing. Nearby web-based users interested in testing could initiate one-on-one discussions with our research assistants by tapping and sending a message.

A conspicuous headline about HIV testing and posters of the 2 VCT models were presented on our official Facebook fan page to encourage viewers to visit and initiate one-on-one message discussions with our research assistants.

In addition, recruitment was carried out through the Facebook, Instagram, and X accounts of 6 celebrities, each with an average follower count exceeding 10,000. Information regarding the 2 VCT models was posted and discussed at least twice every month during the research period on their pages. The audience members who were interested in HIV testing could link to our Line account.

Moreover, friend referrals were used. Screening information were sourced from individuals who had either seen our recruitment posters or undergone our screening process and then shared it with their friends. Those friends who had viewed our recruitment information actively participated in one-on-one discussions with the research assistants through our official account on any social media platform.

#### Grouping Condition

The 2 VCT model options were discussed through messages on each platform. Mobile VCT was provided to participants who lived in the Taipei area and were available for face-to-face meetings. Video VCT was provided to participants who lived in the Taipei area but were not available for face-to-face meetings owing to the social and physical distancing mandates of COVID-19 prevention, and for those who lived in remote areas distant from Taipei.

#### Mobile Voluntary HIV Counseling and Testing Model

Well-trained research assistants delivered the mobile VCT at the time and place designated for the participants. Protective equipment against COVID-19 infection, such as masks, gloves, and goggles, were used by the research assistants when conducting the mobile VCT. The location choices were based on convenience and had compartmentalized seating, such as a convenience store, fast food restaurant, coffee shop, or park. After checking the participants’ identity through the messaging record on their mobile phones, participants were asked to complete the questionnaires by following a URL. Pretest counseling was provided and a blood test was performed by a skin prick using a rapid HIV self-testing kit. The results were interpreted immediately upon completion of the test, followed by posttest counseling and resource referrals. In addition, we accompanied the participants who tested positive to AIDS-designated hospitals for confirmatory diagnosis and CD4 testing.

#### Video Voluntary HIV Counseling and Testing Model

**Figure 1:**
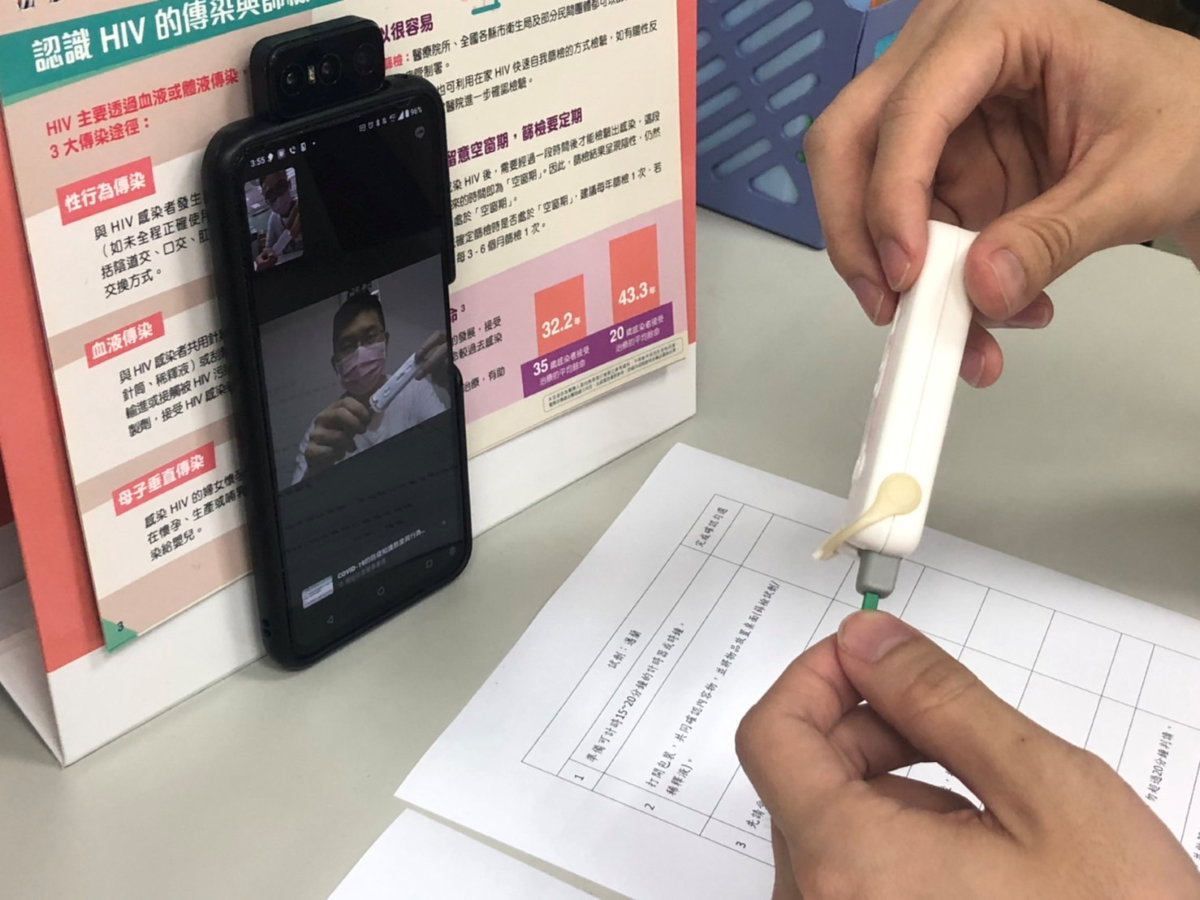
Demonstration of video voluntary HIV counseling and testing in Taiwan during the COVID-19 pandemic.

A rapid HIV self-testing kit was promptly and anonymously dispatched to participants’ designated addresses using a password-protected system. Subsequently, a research assistant made a video call through the Line app to the participant at the appointed time and place, such as at home, at a coffee shop, or in a private location. Participants’ identities were confirmed by verifying the messaging record and box number of the testing kits presented by the screen. They were provided a URL for research consent, where participants can click to agree to join the study and then complete the questionnaire. To maintain privacy, participants could choose only to display their hands-on testing process on the camera screen. Next, participants received pretest counseling before using a rapid HIV self-testing kit to perform the test. They obtained a blood drop by a skin prick and followed instructions provided by the research assistant (Figure 1). The results were displayed on the video screen and confirmed by the research assistant. Posttest counseling and resource referral sections mirrored those in the mobile VCT model. 

### Instrument

#### Demographic Characteristics

The participants’ demographic information, including age, sex, sexual orientation, marital status, education level, and presence of stable income, were collected using a questionnaire. Furthermore, the questionnaire inquired about the source of recruitment information leading to the present test and testing location.

#### HIV-Related Risk-Taking and Protective Behavior

Based on previous studies in Taiwan [37,44], this study inquired about high-risk and preventive behavior for HIV, such as preferred sexual position, meeting sexual partners through social media, having multiple sexual partners, number of sexual partners in the past 3 months, frequency of condom use during recent sexual intercourse, history of regular HIV testing, testing frequency in the preceding year, engagement in sex work, history of sexually transmitted diseases (STDs), recreational drug use in the past 3 months, and previous use of pre-exposure prophylaxis (PrEP) and postexposure prophylaxis (PEP).

#### Rapid HIV Self-Testing Kits

This study used 2 rapid HIV self-testing kits, namely SURE CHECK HIV 1/2 Assay (Chembio) and Mylan HIV Self-Test (Mylan). These kits were approved by the Taiwan Food and Drug Administration (procurement identification 1109042028A and 1109042028B). These tests yield results in approximately 15 minutes and have high sensitivity (95.2% to 97%) and specificity (99.6% to 100%).

#### Cost

This study recorded the consumables and associated administrative expenses used for each VCT session, including test kits, transportation expenses, personnel salaries, internet charges, and the COVID-19 protective equipment for mobile VCT; and test kits, postage, personnel salaries, internet charges, and video devices for video VCT. The total cost was calculated and divided by the average number of participants to determine the average cost per VCT session.

### Statistical Analyses

The recruitment and completion rates for both VCT models were calculated as percentages. Geographic coverage was assessed by evaluating the area and participant density, represented as the number of participants per square kilometer. To control for the potential influence of a selection bias, the testing site (Taipei vs non-Taipei area) was adjusted using univariable multinomial logistic regression. The primary outcome comprised HIV testing results, while the secondary outcome included demographic data and data on behavior associated with HIV risk, which were compared between the mobile and video VCT groups.

### Ethical Considerations

The study was approved by the research ethics committee of National Taiwan University (202012HM042). All participants provided consent after receiving a comprehensive explanation of the research details through a web page, including its purpose, methods, participant requirements, risks and benefits, privacy measures, voluntary participation, contact information, data policies, and compensation. Data collection and processing were conducted anonymously. The participants’ privacy was also protected through the use of masks during both VCT models. Upon completion of all procedures, each participant received an electronic gift certificate valued at US $3.27.

## Results

### Recruitment and Completion

Table 1 shows the results of recruitment and completion of 2 VCTs through different recruitment sources. This study generated 5142 responses on social media platforms and friend referrals, and 1187 participants were recruited, with an overall completion rate of 23.08%. Among the participants, 980 (21.8%) out of 4491 and 207 (31.8%) out of 651 completed the mobile and video VCT, respectively.

**Table 1:** Recruitment and completion of the 2 voluntary HIV counseling and testing models for the HIV risk population in Taiwan during the COVID-19 pandemic.

		GSN^a^ dating apps	Facebook	X (Twitter)	Instagram	Line app	Friend referral	Total
**Mobile VCT^b^**								
	Responses, n	1126^c^	1098^d^	785^d^	672^d^	468^e^	342^f^	4491
	One-on-one message discussion, n/N (%)	587/1126 (52.13)	378/1098 (34.43)	240/785 (30.6)	243/672 (36.2)	391/468 (83.6)	287/342 (83.9)	2126/4491 (47.34)
	Acceptance of VCT, n/N (%)	334/587 (56.9)	122/378 (32.3)	76/240 (31.7)	80/243 (32.9)	199/391 (50.9)	169/287 (58.9)	980/2126 (46.1)
	Completion of VCT, n/N (%)	334/1126 (29.66)	122/1098 (11.11)	76/785 (9.7)	80/672 (11.9)	199/468 (42.5)	169/342 (49.4)	980/4491 (21.82)
**Video VCT**								
	Responses, n	177^c^	124^d^	102^d^	103^d^	89^e^	56^f^	651
	One-on-one message discussion, n/N (%)	93/177 (52.5)	56/124 (45.2)	44/102 (43.1)	44/103 (42.7)	71/89 (79)	48/56 (85)	356/651 (54.7)
	Acceptance of VCT, n/N (%)	64/93 (68)	25/56 (44)	18/44 (40)	18/44 (40)	46/71 (64)	36/48 (75)	207/356 (58.2)
	Completion of VCT, n/N (%)	64/177 (36.2)	25/124 (20.2)	18/102 (17.6)	18/103 (17.5)	46/89 (51)	36/56 (64)	207/651 (31.8)

^a^GSN: geosocial network.

^b^VCT: voluntary counseling and testing.

^c^Number of users who tapped on our official account on Hornet, Grindr, and Gsland.

^d^Number of viewers on the recruitment information page of internet celebrities’ pages on Facebook, X (Twitter), and Instagram, as well as our Facebook fan page.

^e^Number of internet users who actively searched for and browsed the internet to find our official Line account and QR code and requested to be added as friends through the Line app.

^f^Referred by friends who have either read our recruitment information or used our VCT service before.

Responses for mobile VCT comprised 1126 on GSN dating apps, 1098 on Facebook, 785 on X, 672 on Instagram, and 468 on Line, as well as 342 responses through friend referrals. The completion rate was 21.82% (980/4491). Among the recruitment sources, friend referral had the highest completion rate of mobile VCT (49.4%, 169/342), followed by Line (42.5%, 199/468) and GSN dating apps (29.66%, 334/1126). The main reasons for not completing mobile VCT after one-on-one discussion were not meeting the inclusion criteria (15.01%, 172/1146), no response (36.47%, 418/1146), concern about COVID-19 infection when going outside (16.58%, 190/1146), preferring to wait beyond the window period of HIV infection in anticipation of obtaining more accurate results (12.65%, 145/1146), feeling unprepared for the test result (8.29%, 95/1146), and missing the appointment without an explanation (3.32%, 38/1146).

The overall completion rate of video VCT was 31.8% (207/651). Friend referral exhibited the highest completion rate (64%, 36/56), followed by Line (52%, 46/89) and GSN dating apps (36.2%, 64/177). The main reasons for not completing video VCT after one-on-one discussion were not meeting the inclusion criteria (13.4%, 20/149), no response (33.6%, 50/149), not trusting self-testing and wishing to have blood collected by a well-trained person (14.8%, 22/149), fear of drawing blood (12.8%, 19/149), feeling unprepared for the test result (8.1%, 12/149), concern that receiving the parcel may expose their privacy (7.4%, 11/149), and missing the appointment without an explanation (2.7%, 4/149).

### Geographic Distribution

Among the participants in the mobile and video VCT groups, 100% (980/980) and 75.4% (156/207), respectively, lived in Taipei, and 24.6% (51/207) of the participants in the video VCT group lived in remote areas outside Taipei.

The total area covered by the mobile VCT was 390.2 km^2^, with a participant density of 2.51 (980/390.2 km^2^). This covered 1.08% of Taiwan’s total land area (36,197.0 km^2^). The video VCT covered 18 cities and counties, with 24.6% (51/207) of the participants residing in 16 cities and counties far from Taipei, where the mobile VCT did not reach. The coverage area of the video VCT was 26,391.04 km^2^, with a participant density of 0.008 (207/26391.04 km^2^). This covered 72.91% of Taiwan’s total land area.

The participant density for mobile VCT was 313.8 (2.51/0.008 km^2^) times higher compared to video VCT. However, the geographic area covered by the video VCT was 67.6 (26391.04/390.2 km^2^) times larger than that of the mobile VCT.

### Demographic Characteristics

The mean age of all participants was 30.59 (SD 6.7) years. The majority were men (1084/1187, 91.3%), identified as homosexual (855/1187, 72%), and reported being unmarried (1158/1187, 97.6%). In addition, most participants held a college or university-level education (837/1187, 70.5%) and had a stable income (1023/1187, 86.2%). The analysis of demographic characteristics indicated homogeneity between the 2 groups (Table 2).

**Table 2:** The comparison of demographic data between the 2 VCT^a^ models in Taiwan during the COVID-19 pandemic (adjusted for the testing site in the Taipei area and non-Taipei area).

Variable			Total (N=1187), n (%)		Mobile VCT model (n=980), n (%)		Video VCT model (n=207), n (%)		OR^b^ (95% CI)		*P* value
**Age group (year)**											
	<30 (reference)	558 (47)		462 (47.1)		96 (46.4)		—^c^		—	
	≥30	629 (53)		518 (52.9)		111 (53.6)		0.97 (0.72-1.31)		.84	
**Sex**											
	Female (reference)	103 (8.7)		87 (8.9)		16 (7.7)		—		—	
	Male	1084 (91.3)		893 (91.1)		191 (92.3)		0.86 (0.49-1.50)		.59	
**Sexual orientation**											
	Others^d^ (reference)	26 (2.2)		24 (2.4)		2 (1)		—		—	
	Homosexual	855 (72)		700 (71.4)		155 (74.9)		0.38 (0.09-1.61)		.19	
	Heterosexual	126 (10.6)		106 (10.8)		20 (9.7)		0.44 (0.10-2.02)		.29	
	Bisexual	180 (15.2)		150 (15.3)		30 (14.5)		0.42 (0.09-1.86)		.25	
**Marital status**											
	Yes (reference)	29 (2.4)		23 (2.3)		6 (2.9)		—		—	
	No	1158 (97.6)		957 (97.7)		201 (97.1)		1.24 (0.50-3.09)		.64	
**Education**											
	High school or less (reference)	105 (8.8)		89 (9.1)		16 (7.7)		—		—	
	University or college	837 (70.5)		687 (70.1)		150 (72.5)		0.82 (0.47-1.44)		.50	
	Above university	245 (20.6)		204 (20.8)		41 (19.8)		0.89 (0.45-1.68)		.73	
**Stable income**											
	No (reference)	164 (13.8)		137 (14)		27 (13)		—		—	
	Yes	1023 (86.2)		843 (86)		180 (87)		0.92 (0.59-1.44)		.72	

^a^VCT: voluntary counseling and testing.

^b^OR: odds ratio.

^c^Not applicable.

^d^Included pansexuality and uncertainty.

### Risk-Taking and Protective Behavior and Test Results

The majority of participants reported having a preference for both top and bottom sexual positions (455/1187, 38.3%), meeting sexual partners through social media (1003/1187, 84.5%), having 2-3 sexual partners (461/1187, 54.9%), inconsistently using condoms in the past 3 months (952/1187, 80.2%), and not having an HIV test regularly within the previous year (678/1187, 57.1%). Some participants reported a history of sex work, STDs, and recreational drug use in the past 3 months. Most participants had not used PrEP or PEP (950/1187, 80%; Table 3).

**Table 3:** The comparison of risk-taking features between the 2 voluntary HIV counseling and testing models in Taiwan during the COVID-19 pandemic (adjusted for the testing site in the Taipei area and non-Taipei area).

Variable		Total (N=1187), n (%)	Mobile VCT^a^ model (n=980), n (%)	Video VCT model (n=207), n (%)	OR^b^ (95% CI)	*P* value
**Preferred sexual position**						
	Side (reference)	27 (2.3)	26 (2.7)	1 (0.5)	—^c^	—
	Top	386 (32.5)	321 (32.8)	65 (31.4)	0.19 (0.03-1.42)	.11
	Bottom	319 (26.9)	266 (27.1)	53 (25.6)	0.91 (0.02-1.45)	.11
	Both top and bottom	455 (38.3)	367 (37.4)	88 (42.5)	0.16 (0.02-1.20)	.07
**Meeting sex partners through social media**						
	No (reference)	184 (15.5)	166 (16.9)	18 (8.7)	—	—
	Yes	1003 (84.5)	814 (83.1)	189 (91.3)	0.47 (0.28-0.78)	.004
**Multiple sex partners^d^**						
	No (reference)	348 (29.3)	279 (28.5)	69 (33.3)	—	—
	Yes	839 (70.7)	701 (71.5)	138 (66.7)	1.26 (0.91-1.73)	.16
**Number of multiple sexual partners^d^ (n=839)**						
	2-3 (reference)	461 (54.9)	379 (54.1)	82 (59.4)	—	—
	4-9	240 (28.6)	198 (28.2)	42 (30.4)	1.02 (0.68-1.54)	.93
	>10	138 (16.4)	124 (17.7)	14 (10.1)	1.92 (1.05-3.50)	.03
**Frequency of condom usage^d^**						
	Always (reference)	235 (19.8)	185 (18.9)	50 (24.2)	—	—
	Not always	952 (80.2)	795 (81.1)	157 (75.8)	1.01 (0.74-1.36)	.97
**Regularly HIV testing^e^**						
	No (reference)	678 (57.1)	560 (57.1)	118 (57)	—	—
	Yes	509 (42.9)	420 (42.9)	89 (43)	0.99 (0.74-1.35)	.97
**Testing frequency^e^ (n=509)**						
	Per half month to once a year (reference)	230 (45.2)	185 (44)	45 (50.6)	—	—
	Per 3 months	279 (54.8)	235 (56)	44 (49.4)	1.29 (0.82-2.05)	.26
**Experience of sex work**						
	No (reference)	1090 (91.8)	888 (90.6)	202 (97.6)	—	—
	Yes	97 (8.2)	92 (9.4)	5 (2.4)	4.19 (1.68-10.43)	.002
**Experience of STDs^d,f^**						
	No (reference)	1067 (89.9)	871 (88.9)	196 (94.7)	—	—
	Yes	120 (10.1)	109 (11.1)	11 (5.3)	2.23 (1.18-4.23)	.01
**Experiences of recreational drug use^d^**						
	No (reference)	1046 (88.1)	856 (87.3)	190 (91.8)	—	—
	Yes	141 (11.9)	124 (12.7)	17 (8.2)	1.62 (0.95-2.75)	.08
**Adopting PrEP^g^ or PEP^h^**						
	No (reference)	950 (80)	791 (80.7)	159 (76.8)	—	—
	Yes	237 (20)	189 (19.3)	48 (23.2)	0.79 (0.55-1.13)	.20
**HIV testing result**						
	Negative (reference)	1178 (99.2)	973 (99.3)	205 (99)	—	—
	positive	9 (0.8)	7 (0.7)	2 (1)	0.74 (0.15-3.58)	.71

^a^VCT: voluntary counseling and testing.

^b^OR: odds ratio.

^c^Not applicable.

^d^Within the past 3 months.

^e^Within the previous year.

^f^STDs: sexual transmitted diseases.

^g^PrEP: pre-exposure prophylaxis.

^h^PEP: postexposure prophylaxis.

Participants in the video VCT group were more likely to meet sexual partners through social media than those in the mobile VCT group. Participants in the mobile VCT group were more likely to have had more than 10 sexual partners, a history of sex work, and STDs in the past 3 months than those in the video VCT group. No statistically significant difference was observed in HIV-positive rates between the 2 groups. The average CD4 count of HIV-positive participants was 347/μL in the video VCT model (n=2), which was lower than that in the mobile VCT model (460/μL; n=7).

### Testing Costs

The average cost of the video VCT was approximately US $54.68 per participant, which was slightly higher than that of the mobile VCT (US $50.36).

## Discussion

### Principal Findings

This study recruited participants for 2 VCT models through social media platforms and friend referrals during the COVID-19 pandemic and evaluated the test results. The results demonstrated that recruiting for VCT through friend referral and social media platforms was feasible for reaching people with various HIV-related, risk-taking behaviors and HIV case findings while complying with COVID-19 prevention regulations. Furthermore, friend referral through Line had the best success rate for both VCT models. Our research findings indicate that this approach is highly valuable beyond the context of a pandemic and should be integrated into the current screening model to enhance HIV case findings.

The sample size was asymmetric for the 2 VCT models, with 4.7 times more participants opting for mobile than video VCT. Furthermore, most of the participants lived in Taipei. This may be because Taipei and New Taipei have the highest prevalence of HIV in Taiwan [45], creating a higher demand for screening of the HIV risk population in these cities than in other cities and remote areas.

The completion rate of mobile VCT in this study was higher than that reported in 2018 (20.71%), before the COVID-19 pandemic [17]. This increase may be attributed to the requirement to show identification to enter medical facilities for HIV testing during the COVID-19 pandemic, which discouraged individuals who preferred anonymous screening and led them to turn to mobile VCT. Mobile VCT could be performed anonymously outdoors, reducing the risk of COVID-19 transmission associated with entering crowded areas. Furthermore, eligible participants were informed during one-on-one message discussions that the screening personnel tested negative for COVID-19 and did not have any relevant symptoms; protective gear would be used while conducting HIV testing; and they could meet in an open and not crowded location, such as a park. In addition, the discussion dispelled common rumors regarding COVID-19, such as the possibility of COVID-19 transmission through mobile phones and that anti-HIV medication can prevent COVID-19 infection [46]. The mobile VCT was free and anonymous, provided timely and appropriate service, and disseminated accurate COVID-19 prevention information before the test; therefore, it promoted acceptance.

The video VCT model had an overall complete rate of 31.8%, which was higher than that of the mobile VCT. Previous studies before the COVID-19 pandemic recruited participants through a public website and Facebook advertisements and mailed at-home, self-testing kits for HIV and STDs to the participants; these studies reported return rates of 27.2% and 43.7%, respectively [23,44]. The present results for video VCT were consistent with these findings. People may have become aware of the limited HIV testing resources owing to social and physical distancing mandates and visitor control in hospital and health care facilities. In addition, some individuals resided out of range of mobile VCT. Therefore, participants who met the criteria for video VCT had high motivation to accept it. The advantages of video VCT were not only the ability to avoid personal information exposure but also the ability to receive individualized and visible guidance and immediate emotional support in a stigma-free environment from a well-trained research assistant. This result echoes the advantages of video intervention, including the lack of geographical restrictions for delivery, convenience, saving travel time, and reducing the risk of COVID-19 exposure and transmission [20,24,27,47-49]. However, the disadvantage is the decreased results of video interactions if the network speed is unstable or screen quality is poor. Furthermore, this study encountered 5 testing errors, including 3 unsuccessful blood collections by the participants and 2 incorrect results. The participants may feel distressed while waiting for new testing packages.

The completion rates for the 2 VCT models following recruitment through social media platforms, including X, Facebook, and Instagram, were lower than those of friend referral, Line, and GSN apps. Previous studies have found a considerable amount of misleading information spread through social networks during the COVID-19 pandemic, leading to unhealthy behaviors and psychological stress [50,51]. This could potentially divert users’ attention away from HIV screening information. Unlike social media platforms, Line and GSN app interfaces do not contain excessive shared photos and posts on the COVID-19 pandemic and daily life information to divert users’ attention. This may make it easier to enter one-on-one message discussion and complete HIV screening. Furthermore, referrals from friends, particularly those who had used VCT services and could attest to their privacy and credibility, through Line, had the highest completion rate among the channels used in this study [52]. Compared with urban areas, people in rural areas have limited HIV testing resources and experience more difficulty accessing testing owing to transportation to the screening location being inconvenient and distrust toward health care providers [53,54]. In this study, video VCT covered a larger geographic area than mobile VCT and expanded to remote cities and counties. Sharing of recruitment messages on social media platforms by internet celebrities and friend referral play a critical role in disseminating trustable information to areas with limited screening resources and offering additional testing options [17,55,56].

Participants recruited through the 2 models exhibited differences in risk-taking behaviors. The video VCT model required 4-7 days to complete, including distributing the screening kit and conducting testing. Conversely, the mobile VCT provided real-time service within 1-2 days of initial consultation and flexible test time and location. Individuals with a high frequency of risk-taking behaviors and immediate demand for HIV testing [57,58] were more likely to use the mobile VCT model. A previous study revealed a significantly positive correlation between meeting sexual partners through social media and seeking online sexual health–related information [59]. For internet users, the operation of video VCT is simple and familiar [60]. This could explain our results that participants who met sexual partners through social media may have more opportunities to receive recruitment information and use video VCT.

The newly identified HIV-positive rate was 1% in the video VCT group and 0.7% in the mobile VCT group, surpassing the 0.5% rate recorded for mobile VCT in 2020 (program CW108056), before the COVID-19 pandemic. These findings align with previous research findings indicating an elevated HIV-positive rate in MSM during the COVID-19 pandemic [7,8]. This may be because participants in both VCT groups were mostly MSM and their risk-taking behaviors, which included mainly having multiple sexual partners, not always using condoms, and recreationally using drugs, persisted and increased during the COVID-19 pandemic [12,13]. The average CD4 count of participants who are HIV positive in the video VCT group was lower than that in the mobile VCT group and reached the standard of a late diagnosis (<349/μL) [61]. A previous study demonstrated that the late diagnosis rate of HIV increased during the COVID-19 pandemic [62]. Further research using more extensive datasets is required to determine whether video VCT improves late diagnosis of HIV, particularly in remote areas.

The average costs of the 2 models in this study were significantly higher than the cost of self-screening using a kit purchased through a vending machine (US $33.81), without pre- and posttest counseling [63]. The higher costs in this study were mainly due to personnel costs. Considering the importance of one-on-one consultations, all positive cases identified through initial screening in this study were successfully referred to hospital case managers for a confirmed diagnosis and further treatment. Future studies should investigate the effectiveness of personal counseling on participants, focus on enhancing HIV prevention knowledge and behaviors, emotional regulation, reduced COVID-19 infections, and evaluating service aspects such as usability, performance, and satisfaction.

### Limitations

This study had several limitations. First, the use of a cross-sectional design precluded identification of causal relationships between the independent and dependent variables. Second, as this study used a questionnaire survey to collect data, participants may have provided socially expected responses, thus affecting the validity of the research outcomes. Third, owing to the specific recruitment conditions, the study results can only be generalized to participants who completed the whole process. Fourth, the applicability of the study findings is limited to countries and regions characterized by high mobile phone usage and internet connectivity. Fifth, the recruitment may not have effectively reached HIV risk population in remote areas. Future studies could examine undiscovered social media platforms used by HIV risk population in remote areas to enhance the accessibility of video screening information and recruitment. Finally, the high cost of this study warrants further investigation of the cost-effectiveness and feasibility of this design.

### Conclusions

This study demonstrated the feasibility of recruiting participants for HIV screening through social media platforms, particularly by one-on-one message discussions through instant messaging facilitated by friend referrals. Furthermore, the findings revealed the efficacy of continuing VCT for HIV during restrictions associated with the COVID-19 pandemic. Our findings suggest that mobile and video VCT can provide accessible screening options tailored to the testing needs of at-risk groups, thereby promoting their testing motivation and maintaining HIV detection during public health emergencies, such as the COVID-19 pandemic. The findings of this study provide valuable insights for enhancing HIV screening strategies amid emerging infectious disease pandemics.

## Abbreviations

GSN: geosocial network
LGBTQ: lesbian, gay, bisexual, transgender, and queer
MSM: men who have sex with men
OR: odds ratio
PEP: postexposure prophylaxis
PrEP: pre-exposure prophylaxis
STD: sexually transmitted disease
VCT: voluntary counseling and testing

## Data Availability

The analyzed data are unavailable owing to the privacy policy of the research ethics committee of this study.
